# Glycoside hydrolase gene transcription by *Alicyclobacillus acidocaldarius* during growth on wheat arabinoxylan and monosaccharides: a proposed xylan hydrolysis mechanism

**DOI:** 10.1186/s13068-018-1110-3

**Published:** 2018-04-16

**Authors:** Brady D. Lee, William A. Apel, Peter P. Sheridan, Linda C. DeVeaux

**Affiliations:** 1 Biological Systems Department, Idaho National Laboratory, P. O. Box 1625, Idaho Falls, ID 83415 USA; 2 Department of Biological Sciences, Idaho State University, Campus Box 8007, Pocatello, ID 83209 USA; 3 Department of Biology, New Mexico Institute of Mining and Technology, 801 Leroy Pl, Socorro, NM 87801 USA; 4 Present Address: Pacific Northwest National Laboratory, Energy and Environment Directorate, Richland, WA USA

**Keywords:** *Alicyclobacillus acidocaldarius*, Wheat arabinoxylan, Glycoside hydrolase, Microarray

## Abstract

**Background:**

Metabolism of carbon bound in wheat arabinoxylan (WAX) polysaccharides by bacteria requires a number of glycoside hydrolases active toward different bonds between sugars and other molecules. *Alicyclobacillus acidocaldarius* is a Gram-positive thermoacidophilic bacterium capable of growth on a variety of mono-, di-, oligo-, and polysaccharides. Nineteen proposed glycoside hydrolases have been annotated in the *A. acidocaldarius* Type Strain ATCC27009/DSM 446 genome. Experiments were performed to understand the effect of monosaccharides on gene expression during growth on the polysaccharide, WAX.

**Results:**

Molecular analysis using high-density oligonucleotide microarrays was performed on *A. acidocaldarius* strain ATCC27009 when growing on WAX. When a culture growing exponentially at the expense of arabinoxylan saccharides was challenged with glucose or xylose, most glycoside hydrolases were downregulated. Interestingly, regulation was more intense when xylose was added to the culture than when glucose was added, showing a clear departure from classical carbon catabolite repression demonstrated by many Gram-positive bacteria. In silico analyses of the regulated glycoside hydrolases, along with the results from the microarray analyses, yielded a potential mechanism for arabinoxylan metabolism by *A. acidocaldarius*. Glycoside hydrolases expressed by this strain may have broad substrate specificity, and initial hydrolysis is catalyzed by an extracellular xylanase, while subsequent steps are likely performed inside the growing cell.

**Conclusions:**

Glycoside hydrolases, for the most part, appear to be found in clusters, throughout the *A. acidocaldarius* genome. Not all of the glycoside hydrolase genes found at loci within these clusters were regulated during the experiment, indicating that a specific subset of the 19 glycoside hydrolase genes found in *A. acidocaldarius* were used during metabolism of WAX. While specific functions of the glycoside hydrolases were not tested as part of the research discussed, many of the glycoside hydrolases found in the *A. acidocaldarius* Type Strain appear to have a broader substrate range than that represented by the glycoside hydrolase family in which the enzymes were categorized.

## Background

*Alicyclobacillus acidocaldarius* is a Gram-positive, spore-forming, thermophilic acidophile that grows optimally in strictly aerobic conditions at 60 °C and at pH levels between 3 and 4. This bacterium was isolated from a hot spring in Nymph Creek area of Yellowstone National Park [1]. Originally classified as *Bacillus acidocaldarius*, it was reclassified as *A. acidocaldarius* based on the prevalence of ω-cyclic fatty acids in the cell wall and an abbreviated helix 6 of the 16S rRNA [2]. *A. acidocaldarius* has subsequently been isolated from diverse habitats, including water and soil from geothermal sites, submarine hot springs, and orchard soils, and also as a contaminant in heat-processed foods (e.g., fruit juices) [3–8]. *A. acidocaldarius* has demonstrated the ability to gain cellular carbon and energy from a wide variety of 5- and 6-carbon sugars, including l-arabinose, ribose, d-xylose, d-galactose, d-fructose, d-mannose, rhamnose, mannitol, and tagatose; the disaccharides d-turanose, melibiose, cellobiose, lactose, maltose, sucrose, and trehalose, as well as the more complex polysaccharides: cellulose, hemicellulose (xylan), starch, and glycogen [9].

Lignocellulosic biomass is currently being studied as a feedstock for producing fuels and chemicals [10, 11]. Chemically, lignocellulose is a heterogeneous, three-dimensional matrix made of carbohydrate, primarily hemicellulose (20–30%) and cellulose (40–50%), and lignin. These components are intertwined, providing a structure that is highly resistant to microbial degradation. The hemicellulose fraction accounts for nearly one-third of the renewable carbon found on Earth [12, 13]. Hemicellulose is composed predominantly of xylan, which consists of a conserved backbone of 1,4-linked β-d-xylose residues, decorated with side groups composed of arabinose, glucuronic acid, 4-*O*-methyl-glucuronic acid, and sometimes galactose [14].

A thorough understanding of polysaccharide, monosaccharide, and mixed saccharide metabolism by *A. acidocaldarius* will help determine the utility of this bacterium for lignocellulose depolymerization. In part, this can be accomplished by understanding the effect of transcriptional regulation [i.e., carbon catabolite repression (CCR) as one example] on expression of genes encoding glycoside hydrolases, which represents the first step in metabolism of lignocellulose. Genome analysis of *A. acidocaldarius* has revealed the presence of all components of Gram-positive CCR, as well as other regulators of genes encoding enzymes for monosaccharide and polysaccharide metabolism. The purpose of this study was to monitor gene transcription during growth on the polysaccharide, WAX, and to monitor the regulatory effects of monosaccharides on transcription of *A. acidocaldarius* genes related to xylan use (i.e., glycoside hydrolases). Analyses were performed using batch chemostat studies and global transcriptome analysis using high-density oligonucleotide microarray studies. This research represents the first transcriptome analysis of *A. acidocaldarius* growing on plant polysaccharides, such as WAX.

## Results

### Glycoside hydrolase inventory of *A. acidocaldarius*

*Alicyclobacillus acidocaldarius* represents a source of thermostable glycoside hydrolases for application as industrial catalysts for the hydrolysis of the cellulose and hemicellulose components of lignocellulose. To date, 19 glycoside hydrolase genes have been identified in the *A. acidocaldarius* genome [15], and β-galactosidase, α-amylase, cellulase, neopullulanase, exo-pectinase, mannanase, β-glycosidase, and endoglucanase enzymes have been expressed and characterized [6, 9, 16–33]. Enzymes required for efficient depolymerization of lignocellulose, and glycoside hydrolase activities found encoded in the genome of *A. acidocaldarius* or have been characterized by other research laboratories using recombinant *A. acidocaldarius* enzymes, are shown (Table 1). Analysis of the *A. acidocaldarius* genome shows 19 gene loci that are annotated as glycoside hydrolases (EC 3.2.1.-), supporting the observed broad substrate range of *A. acidocaldarius* related to oligo- and polysaccharides for this bacterium (Table 2). Only three of the loci (Aaci_0048, Aaci_2457, and Aaci_2874) encode proteins that contain a signal peptide, which would indicate extracellular activity. The remaining enzymes are likely located and active in the periplasm or cytoplasm of *A. acidocaldarius*.

**Table 1:** Glycoside hydrolase genes required for complete depolymerization of the cellulose and hemicellulose fractions of lignocellulose and enzymes encoded by genes in the *A. acidocaldarius* genome

Glycosyl hydrolase activity required for lignocellulose depolymerization	Presence in *A. acidocaldarius*
Cellulose	
Endo-β-1,4-glucanase (EC 3.2.1.4)	X
1,4-β-glucan glucanohydrolase (EC 3.2.1.74)	
1,4-β-glucan cellobiohydrolase (EC 3.2.1.91)	
β-Glucosidase (EC 3.2.1.21)	X
Hemicellulose	
Endo-β-1,4-xylanase (EC 3.2.1.8)	X
Exo-β-1,4-xylosidase or β-xylosidase (EC 3.2.1.37)	X
α-l-arabinofuranosidase (EC 3.2.1.55)	X
Endo-α-1,5-arabinanase (EC 3.2.1.99)	
α-Glucuronidase (EC 3.2.1.139)	X
Endo-β-1,4-mannanase (EC 3.2.1.78)	
Exo-β-1,4-mannosidase (EC 3.2.1.25)	
α-Galactosidase (EC 3.2.1.22)	
β-Glucosidase (EC 3.2.1.21)	X
Endo-β-1,4-galactanase (EC 3.2.1.89)	
Acetylxylan esterase (EC 3.1.1.72)	X
Acetylmannan esterase (EC 3.1.1.6)	
Ferulic and *p*-coumaric acid esterases (EC 3.1.1.73)	X

**Table 2:** Identification of genome loci in *A. acidocaldarius* strain DSM 446 genome that encodes putative glycoside hydrolases

Gene locus	Product name	Enzyme Commission number	Family	Signal sequence	Transmembrane helices
Aaci_0048	α-l-arabinofuranosidase-like protein			Yes (24/25)	Yes
Aaci_0060	Xylan α-1,2-glucuronosidase	EC:3.2.1.131	67	No	No
Aaci_0332	β-Glucosidase	EC:3.2.1.21	3	No	No
Aaci_0786	α-Galactosidase	EC:3.2.1.22	36	No	No
Aaci_0789	β-1,4-mannanase	EC:3.2.1.78	113	No	No
Aaci_0797	α-Galactosidase	EC:3.2.1.22	4	No	No
Aaci_0912	Amylo-α-1,6-glucosidase	EC:3.2.1.33	13	No	No
Aaci_1218	β-Galactosidase	EC:3.2.1.23	2	No	No
Aaci_1895	β-Galactosidase/β-glucosidase	EC:3.2.1.21	1	No	No
Aaci_2328	Endo-1,4-β-xylanase	EC:3.2.1.8	10	No	No
Aaci_2457	Polygalacturonase	EC:3.2.1.15	28	Yes (33/34)	Yes
Aaci_2475	Endoglucanase C	EC:3.2.1.4	9	No	No
Aaci_2630	β-Glucosidase/β-xylosidase	EC:3.2.1.21 EC:3.2.1.37	3	No	No
Aaci_2868	α-Glucosidase	EC:3.2.1.20	31	No	No
Aaci_2869	Neopullulanase/cyclomaltodextrinase/maltogenic α-amylase	EC:3.2.1.135 EC:3.2.1.54 EC:3.2.1.133	13	No	No
Aaci_2874	α-Amylase	EC:3.2.1.1	13	Yes (23/24)	No
Aaci_2887	α-Xylosidase	EC:3.2.1.177	31	No	No
Aaci_2891	β-Galactosidase	EC:3.2.1.23	42	No	No
Aaci_2894	α-l-arabinofuranosidase	EC:3.2.1.55	51	No	No

Classification of Enzyme Commission and glycoside hydrolase family numbers were obtained from the Carbohydrate-Active enZYme database (CAZY). Signal sequences and transmembrane helices were taken from JGI-IMG, and signal sequences were verified using the SignalP program

Extent of regulation of each of these genes during the experiment is presented, followed by an in-depth discussion of the bioinformatic analysis to assign function of the enzyme product from these genes. Microarray data discussed in this publication have been deposited in the National Center for Biotechnology Information (NCBI) Gene Expression Omnibus (GEO) and are accessible through GEO Series Accession Number GSE89078.

### Regulation of proposed arabinoxylan hydrolysis genes

**Fig. 1:**
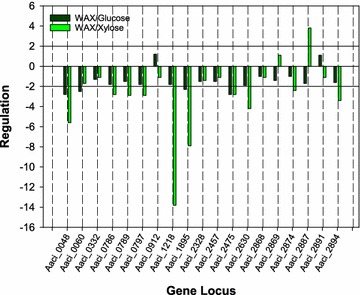
Regulation of glycoside hydrolase genes in *A. acidocaldarius* when grown on WAX alone compared to regulation when growing on WAX and either glucose or xylose. See Table 2 for annotation of genes listed on *x*-axis

Gene transcription by *A. acidocaldarius* grown to mid-exponential phase on WAX was compared to transcription following the addition of glucose or xylose. Following initial exposure of the *A. acidocaldarius* growing on WAX to the monomer sugars, transcription of 13 of the 19 genes that encode putative glycoside hydrolases were downregulated, regardless of whether glucose or xylose was added (Fig. 1). In general, downregulation of glycoside hydrolase gene transcription was greater when xylose was added to the culture than when glucose was added. This is puzzling because in most Gram-positive bacteria, the opposite has been shown [34, 35]. Two glycoside hydrolase genes were upregulated when glucose was added (Aaci_0912 and Aaci_2891), while these same genes were downregulated when xylose was added. Aaci_0912, which is a proposed amylo-α-1,6-glycosidase, was upregulated slightly over onefold (or a 2 times increase comparing final to initial gene transcription) when glucose was added. Since this locus was downregulated when xylose was added, the gene was being expressed at low levels when *A. acidocaldarius* was growing on WAX. Similarly, Aaci_2891, which encodes a β-galactosidase, was positively regulated when glucose was added, but downregulated when xylose was added. Again, regulation of Aaci_2891 was approximately onefold, regardless of sugar or direction of regulation. Two other glycoside hydrolase genes (Aaci_2869 and Aaci_2887) behaved in the opposite manner of the two genes just discussed and were upregulated when xylose was added but downregulated with glucose. One of these genes, Aaci_2887, which is proposed to encode a putative α-xylosidase, was upregulated nearly fourfold with xylose, but was downregulated nearly twofold when glucose was added. The extent of downregulation (1.4-fold) of Aaci_2869 when glucose was added was greater than the magnitude of upregulation on xylose (onefold). Transcription of the remaining 15 glycoside hydrolase genes was downregulated following addition of either xylose or glucose. Gene locus Aaci_0048, which encodes a 959-amino acid protein annotated as a α-l-arabinofuranosidase-like protein, was downregulated 5.6-fold when xylose was added and 2.8-fold when glucose was added. Gene transcription from locus Aaci_0060 from *A. acidocaldarius* was downregulated 2.5-fold when glucose was added and 1.7-fold when xylose was added. The putative protein product is annotated as an α-1,2-glucuronidase, which is most likely involved in hydrolysis of 4-*O*-methyl-d-glucuronic acid or glucuronic acid, both of which are found as modifications of the xylan backbone of WAX. Transcription of gene loci Aaci_0786, Aaci_0789, and Aaci_0797 was downregulated during this experiment, and regulation was greater when xylose was supplied as the inducing sugar. These genes encode a putative α-galactosidase, a hypothetical protein with a glycoside hydrolase catalytic domain, and a second α-galactosidase, respectively. When xylose was added, transcription of gene locus Aaci_1218, a putative β-galactosidase, was downregulated nearly 14-fold, but less than twofold when glucose was added. This gene locus was the most highly downregulated gene proposed to encode a glycoside hydrolase. A second gene (Aaci_1895) that has been annotated as a β-galactosidase/β-glucosidase in glycoside hydrolase family 1 was downregulated 7.9-fold when xylose was added and 2.3-fold when glucose was added. A gene encoding a family 9 glycoside hydrolase (Aaci_2475) was downregulated equally (~ 2.8-fold) when the exponentially growing *A. acidocaldarius* was exposed to xylose or glucose. The transcript for a 782-amino acid protein, encoded by genome locus Aaci_2630, was downregulated nearly twofold when glucose was added and greater than fourfold when xylose was added. This gene encodes a glycoside hydrolase family 3 domain protein with β-glucosidase or β-xylosidase activity. While transcription of genes encoding enzymes that would be active toward glucose polymers would not be expected during growth on a xylan substrate, gene loci Aaci_2874, which encodes a protein annotated as an α-amylase, was downregulated nearly threefold when xylose was added to the exponentially growing culture, but not with glucose. A gene annotated to encode an α-arabinofuranosidase (Aaci_2894) was downregulated 3.4-fold when xylose was added to the logarithmically growing *A. acidocaldarius* culture, but less than twofold when glucose was added. Gene Aaci_2887, annotated as a glycoside hydrolase family 31 enzyme, was upregulated 3.8-fold when xylose was added, but downregulated 1.7-fold when glucose was added.

### Regulation of other accessory genes associated with arabinoxylan hydrolysis

**Fig. 2:**
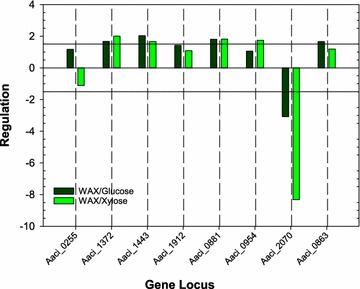
Regulation of carbohydrate deacetylase genes in *A. acidocaldarius* strain DSM 446 when grown on WAX alone compared to regulation when growing on WAX and either glucose or xylose. See Table 3 for annotation of genes listed on *x*-axis

When considering arabinoxylan compounds, the xylose backbone and sugar moieties attached to this backbone can also have acetyl and ferulic acid groups chemically bound in the arabinoxylan structure. For optimal liberation of the sugars in arabinoxylan, a number of carbohydrate esterases (CE) or deacetylase enzymes are also required for removal of these groups. Putative CE enzymes attributed to *A. acidocaldarius* strain DSM 446 as annotated in the CAZy database are listed in Table 3. Typically, CE activity is attributed to families 1–7 and 16, acting on fragments generated by the activity of endo-β-1,4-xylanases [36]. Proteins found in CE family 14, such as LmbE family proteins, have broad substrate specificity and can act as CE [37, 38]. Deacetylation activity by this group of enzymes is primarily associated with cell wall rearrangement and sporulation in bacteria, but the broad substrate specificity may allow these enzymes to act on acetyl moieties found in arabinoxylan or even chitin [39]. Six of the eight esterase-encoding genes were regulated during the experiments (Fig. 2).

**Table 3:** Identification of genome loci in *A. acidocaldarius* strain DSM 446 genome that encodes putative carbohydrate deacetylase enzymes

Gene locus	Product name	Enzyme Commission number	Family	Signal sequence	Transmembrane helices
Aaci_0255	Polysaccharide deacetylase	EC:3.1.1.72 3.5.1.-	4	No	No
Aaci_0863	*N*-acetylglucosamine-6-phosphate deacetylase	EC:3.5.1.25 3.5.1.80	9	No	No
Aaci_0881	LmbE family protein	EC:3.5.1.-	14	No	No
Aaci_0954	LmbE family protein	EC:3.5.1.-	14	No	No
Aaci_1372	Polysaccharide deacetylase	EC:3.1.1.72 3.5.1.-	4	Yes (28/29)	No
Aaci_1443	Polysaccharide deacetylase	EC:3.1.1.72 3.5.1.-	4	No	No
Aaci_1912	Polysaccharide deacetylase	EC:3.1.1.72 3.5.1.-	4	No	No
Aaci_2070	LmbE family protein	EC:3.5.1.-	14	No	No

Classification of Enzyme Commission and carbohydrate deacetylase family numbers were obtained from the Carbohydrate-Active enZYme database (CAZY). Signal sequences and transmembrane helices were taken from JGI-IMG, and signal sequences were verified using the SignalP program

**Fig. 3:**
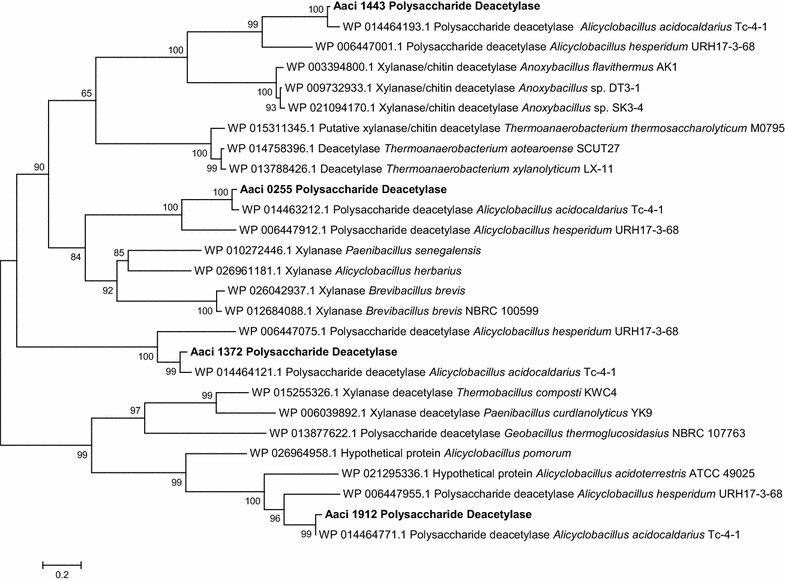
A phylogenetic distance tree showing alignment of CE family 4 deacetylase enzymes and homology to deacetylase enzymes from other bacteria. Each gene in tree is listed by accession number and bacterium carrying gene

Two (Aaci_1372 and Aaci_1443) of the four genes encoding CE family 4 enzymes were upregulated during the experiments. Aaci_1372 was upregulated to a greater extent when xylose was added to the *A. acidocaldarius* strain DSM 446 culture growing on WAX, while the converse was true for Aaci_1443, where greater upregulation was seen when glucose was added. The protein encoded by Aaci_1372 also contains a signal peptide indicating extracellular localization of this enzyme. The protein encoded by Aaci_1372 is homologous to putative xylanase and chitin deacetylase enzymes found in *Desulfosporosinus* species and to polysaccharide deacetylase enzymes in other Alicyclobacilli (Fig. 3). The putative CE family 4 esterase encoded by Aaci_1443 is homologous to polysaccharide deacetylases in other Alicyclobacilli, as well as xylanase/chitin deacetylase enzymes found in a variety of *Anoxybacillus* species. While substrate specificity of the proteins encoded by these two loci has not been determined, upregulation during the experiment and homology to other CE family 4 proteins may indicate that these enzymes are involved in deacetylation of arabinoxylan. One possibility is that these genes were expressed to remove the acetyl groups from small oligosaccharides, allowing further metabolism of the arabinoxylan fractions.

**Fig. 4:**
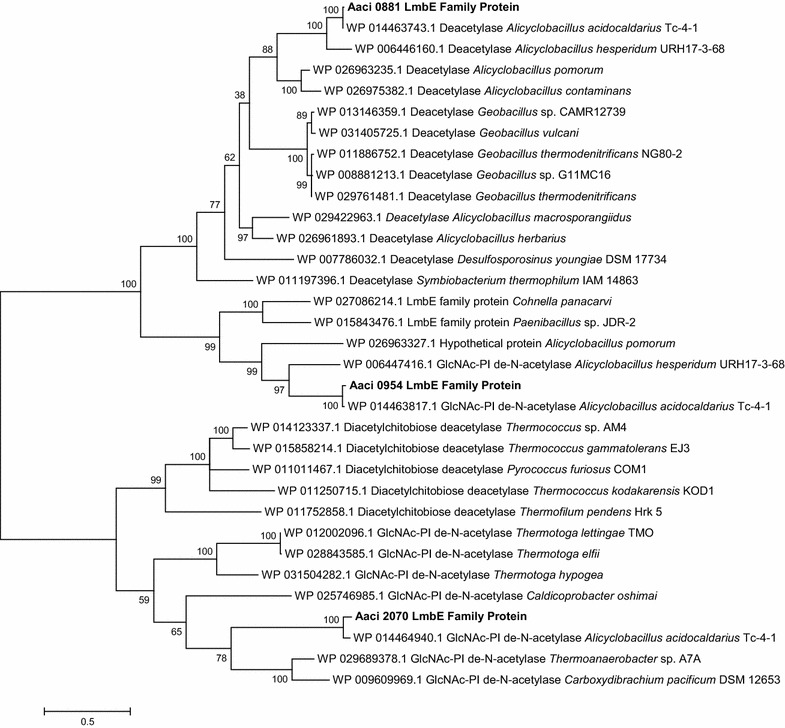
A phylogenetic distance tree showing three LmbE family proteins encoded by gene loci in *A. acidocaldarius* strain DSM 446 and comparison to other *Firmicutes* and hyperthermophiles. Each gene in tree is listed by accession number and bacterium carrying gene

All three genes encoding LmbE family proteins found in the *A. acidocaldarius* strain DSM 446 genome were regulated during the experiments (see Table 3). Aaci_0881 was upregulated to similar levels when either glucose or xylose was added, while Aaci_0954 was only upregulated when xylose was added. In contrast, Aaci_2070 was downregulated during the experiments, 8.3-fold when xylose was added and threefold when glucose was added. Aaci_0881 and Aaci_0954 show homology to annotated deacetylases in other Alicyclobacilli and a number of *Geobacillus* species (Fig. 4). Aaci_2070 shows homology to deacetylases found in many hyperthermophiles. While this group of enzymes has been annotated as deacetylases, their specific function in arabinoxylan depolymerization, if any, is not known.

## Discussion

Lignocellulose has three components—a hexose-containing component (cellulose), a primarily pentose-containing component (hemicellulose), and an aromatic-containing component (lignin)—and accounts for a vast amount of carbon in the Earth’s surface environment [14, 40]. Due to the massive amount of this material in the environment, many microorganisms have developed the ability to hydrolyze the various components into oligomer and monomer units that are further metabolized for cellular processes, or fermented to produce alcohols and organic acids [41]. An important first step in metabolism of lignocellulose is the production of glycoside hydrolases that hydrolyze chemical linkages between the sugars, establishing the secondary structure of the polymeric compounds (i.e., cellulose, hemicellulose, and lignin). The primary objective of the current study was to understand gene transcription during growth on xylan (i.e., hemicellulose) by the Gram-positive thermoacidophile, *A. acidocaldarius*. To accomplish this objective, a physiological, molecular, and in silico approach was carried out to identify the specific glycoside hydrolases active when *A. acidocaldarius* is grown on hemicellulose provided in the form of WAX.

**Fig. 5:**
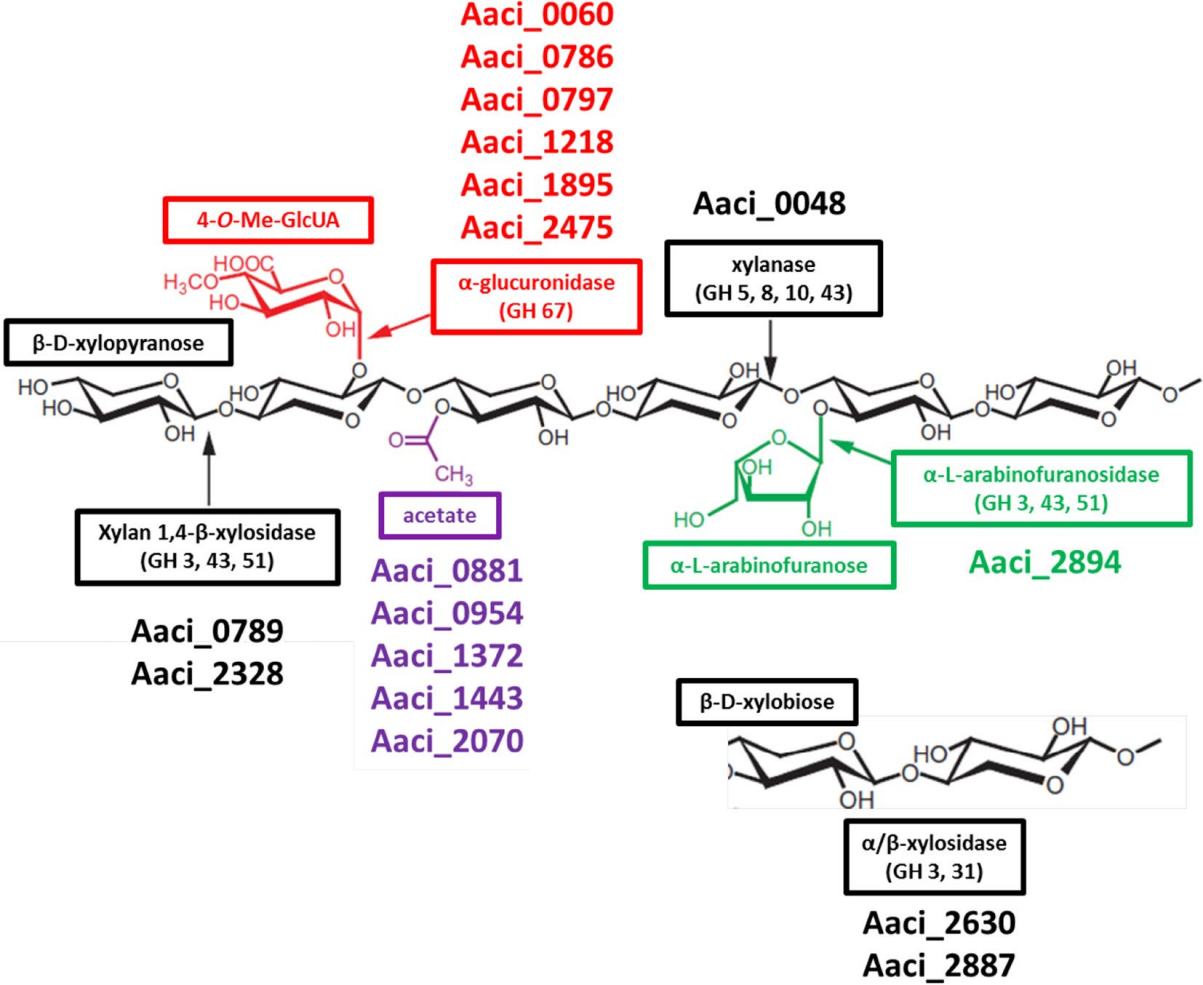
Schematic of arabinoxylan showing chemical structure and proposed *A. acidocaldarius* strain DSM 446 genes annotated to produce enzymes catalyzing hydrolysis of specific bonds in the xylan backbone as well as functional groups attached to the backbone. See Tables 2 and 3 for description of gene products. GH family numbers listed are known to demonstrate common genes associated with hydrolysis of representative bonds, but they do not take into account increased specificity of other enzymes that may hydrolyze these bonds also

Molecular analysis was carried out using high-density oligonucleotide microarray studies, comparing gene expression of *A. acidocaldarius* during logarithmic growth on WAX, to gene expression after a monosaccharide, glucose, or xylose, was spiked into the growth medium. If genes were downregulated when the inducing sugar was added, the assumption was made that the protein product of the gene was being expressed during growth on WAX. Conversely, if genes were upregulated when the monosaccharide was added, the assumption was that the gene product was induced by the sugar. In silico analysis of the glycoside hydrolases expressed by *A. acidocaldarius* was used to determine how xylan metabolism in *A. acidocaldarius* is related to hemicellulose metabolism in other Bacteria and Archaea. Finally, the whole information was combined to propose a model for xylan metabolism by *A. acidocaldarius* (Fig. 5).

Steps in arabinoxylan metabolism hypothesized in this model include an initial extracellular depolymerization, followed by numerous steps inside the cell. The first step in the process is extracellular hydrolysis of β-1,4-bonds between internal xylopyranose residues in the arabinoxylan xylose backbone by the α-arabinofuranosides-like enzyme encoded by Aaci_0048. Studies by Eckert and Schneider have shown xylanase activity by this enzyme [9]. Since the remaining enzymes that were downregulated during the experiment do not contain signal sequences, these enzymes are most likely active in the cytoplasm. Inside the cell, smaller β-d-xylopyranose oligosaccharides, including β-d-xylobiose, would be depolymerized by an endo-acting xylosidase (Aaci_0789 and Aaci_2328) and Aaci_2630 and Aaci_2887 for xylobiose molecules. The upregulation of Aaci_2887 indicates a shift in metabolism to hydrolyze xylobiose residues already inside the cell when the second sugar was added. Removal of α-l-arabinofuranose side groups would then be catalyzed by the α-l-arabinofuranosidase encoded by Aaci_2894. Approximately six enzymes that are annotated for putative activity toward 4-*O*-methyl glucuronic acid or galactose side groups were regulated during the experiments. While galactose is not shown in the schematic (Fig. 5), galactose is often found in arabinoxylan molecules [42]. Finally, a number of proposed esterase enzymes are thought to be active on acetyl- and feruloyl- (not shown in model) groups on the xylan backbone. Polysaccharide deacetylase (Aaci_1372 and Aaci_1443) and LmbE-like proteins (Aaci_0881, Aaci_0952, and Aaci_2070) were regulated during the experiment. Phylogenetic analysis of enzymes encoded by these genes indicated homology to other xylanase type enzymes (Fig. 2) and polysaccharide deacetylase (Fig. 3), which would be involved in removing acetyl side groups from the arabinoxylan molecule.

Bioinformatic analysis comparing the regulated *A. acidocaldarius* glycoside hydrolase genes to other bacteria allowed for putative verification of function for these genes in *A. acidocaldarius*. Comparison of gene locus Aaci_0048, which encodes a 959-amino acid protein annotated as a α-l-arabinofuranosidase-like protein, shows conserved domains from the amino acid sequence are homologous to family 44 glycoside hydrolases, which indicate possible activity toward cellulose and xylan (i.e., endoglucanase activity). The N-terminal domain of the protein also contains a sequence with close homology to carbohydrate binding domains of numerous glycoside hydrolases. Prior to publication of the *A. acidocaldarius* genome, Eckert and Schneider [9] expressed and characterized a 959-amino acid thermophilic endoglucanase (CelB; Accession AJ551527.1) from *A. acidocaldarius* with 100% identity to the gene product from Aaci_0048. The recombinant CelB enzyme demonstrated endo-acting activity toward carboxy methyl cellulose, steam-exploded cellulose and oat spelt xylan, demonstrating a broad substrate range for the enzyme. Sequencing and characterization of the enzyme classified it as an extracellular endoglucanase, with high sequence similarity to GH family 51 of arabinofuranosidases. While this enzyme has been annotated as a α-l-arabinofuranosidase-like protein, no activity was demonstrated using *p*-nitrophenyl-α-l-arabinofuranoside. These results have been verified in unrelated studies at the Idaho National Laboratory for the native enzyme from *A. acidocaldarius* and a recombinant enzyme expressed from *Pichia pastoris* (data not shown). In total, these data indicate that the gene product from Aaci_0048 is a glycoside hydrolase protein that catalyzes hydrolysis of β-1,4-linked xylose subunits in the WAX xylan backbone. It should be noted that the glycoside hydrolase encoded by Aaci_0048 shares 43% homology with a β-1,4-glucanase expressed from *Alicyclobacillus* sp. A4 [43].

**Fig. 6:**
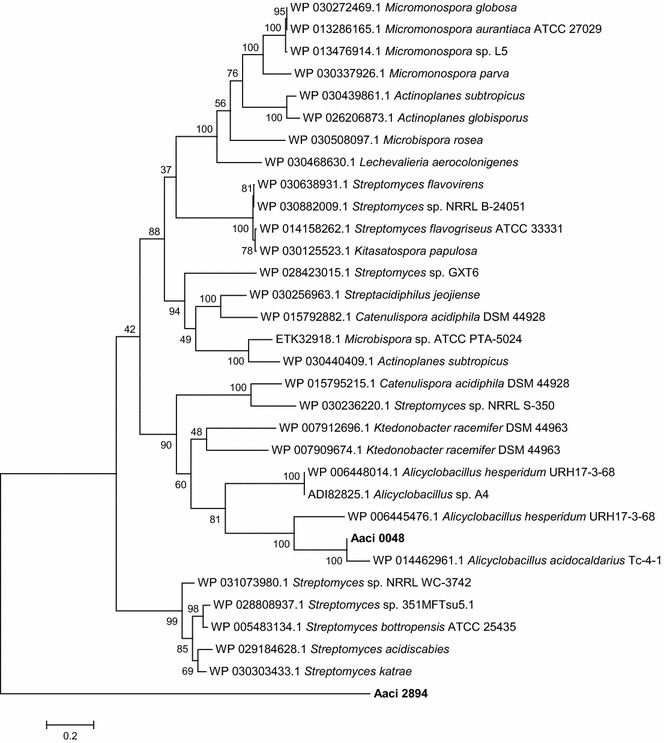
Phylogenetic tree showing distance of α-l-arabinofuranosidase-like protein encoded by gene locus Aaci_0048 in *A. acidocaldarius* strain DSM 446 genome to similarly annotated genes in other Alicyclobacilli and a number of *Actinomycetes*. Each gene in tree is listed by accession number and bacterium carrying gene

The Homology Toolkit of the Integrated Microbial Genomes (IMG) Tool at JGI was used to compare the α-l-arabinofuranosidase-like protein (Aaci_0048) from *A. acidocaldarius* to other genes within IMG. As would be expected, the protein showed the highest percent identity to similarly annotated genes in other species of *Alicyclobacillus*. The most closely related protein sequence from non-*Alicyclobacillus* was a cellulose binding family II protein from *Ktedonobacter racemifer* SOSP1-21. A distance tree generated from sequence information shows that, based on percent identity, Aaci_0048 had the highest homology to α-arabinofuranosidases or cellulose binding family II proteins from numerous species of bacteria from the Order *Actinomycetales* (Fig. 6). Differences in activity between this enzyme and other α-arabinofuranosidases in the database may be due to the additional 200–400 amino acids found in Aaci_0048 and the other similar proteins found in other strains of *A. acidocaldarius*. For comparison, the β-1,4-glucanase expressed from *Alicyclobacillus* sp. A4 still has fewer amino acids than the product of Aaci_0048, which contains an additional ~ 250 amino acids at the C-terminus of the protein sequence. There is very little homology between Aaci_0048 and another annotated α-arabinofuranosidase encoded by Aaci_2894 (discussed below). Evolutionarily, while the exact mechanism of catalysis is not known, an α-arabinofuranosidase could have been transferred from an *Actinomycete*, followed by fusion with a protein that supplied the endoglucanase and endoxylanase activity.

**Fig. 7:**
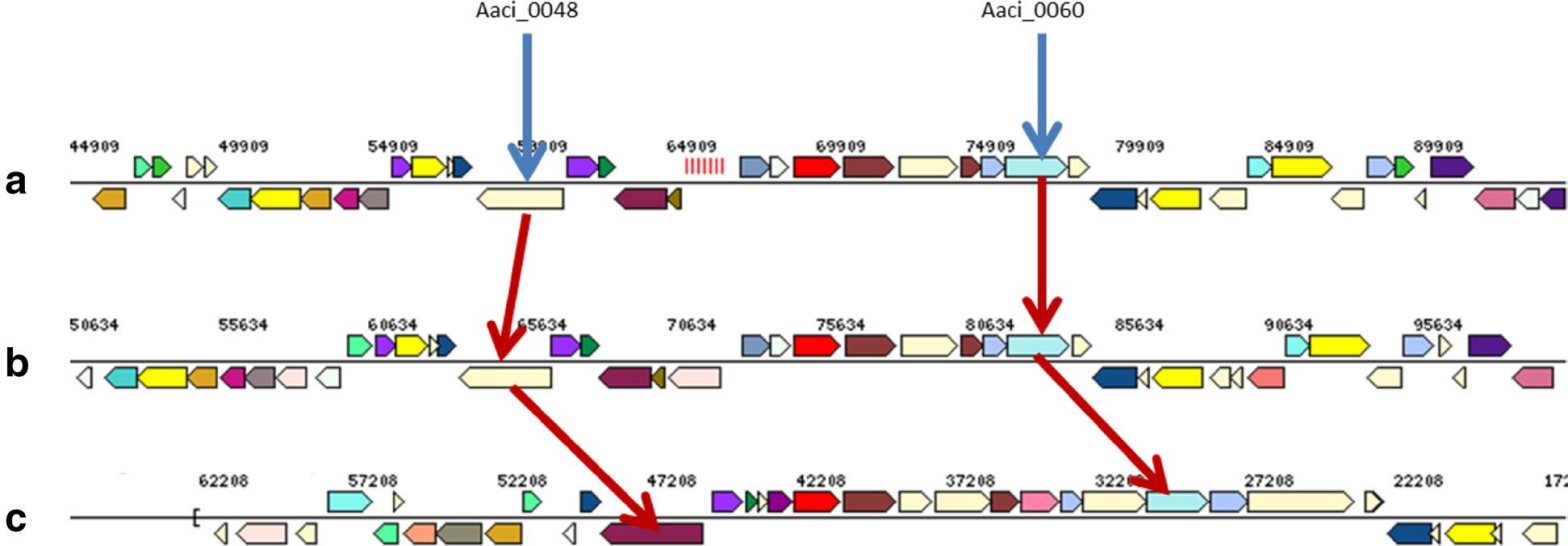
Schematic showing proximity of Aaci_0048 (encoding an α-l-arabinofuranosidase-like protein) and Aaci_0060 (which encodes a xylan α-1,2-glucuronosidase) from *A. acidocaldarius* strain DSM 446 and other *Alicyclobacillus* species. **a** *A. acidocaldarius* strain DSM 446, **b** *A. acidocaldarius* strain Tc-4-1, **c** *A. hesperidium* strain URH17-3-68. Gene neighborhoods were retrieved using the Gene Ortholog Neighborhood Tool within the JGI-IMG database. Blue arrows identify gene loci in *A. acidocaldarius* strain DSM 446, and red arrows identify genes encoding similar proteins in other *Alicyclobacillus* species

The putative protein product from genome locus Aaci_0060 is annotated as an α-1,2-glucuronidase, which is most likely involved in hydrolysis of 4-*O*-methyl-d-glucuronic acid or glucuronic acid, both of which are found as modifications of the xylan backbone of WAX. Multidomain analysis of the protein product of Aaci_0060 supports the annotation as an α-1,2-glucuronidase, and more detailed analysis shows the presence of C-terminal and middle domains (probable catalytic region) found in glycoside hydrolase family 67 proteins [44]. Aaci_0060 and Aaci_0048 may act in concert, since their loci are in the same gene neighborhood of the *A. acidocaldarius* genome; however, the relationship of transcription of these two genes has not been thoroughly studied. The proximity of these two genes appears to be conserved in a variety of *Alicyclobacillus* species, as demonstrated by comparing with the genomes of *A. acidocaldarius* subsp. *acidocaldarius* (Tc-4-1) and *A. hesperidum* URH17-3-68 (Fig. 7).

**Fig. 8:**
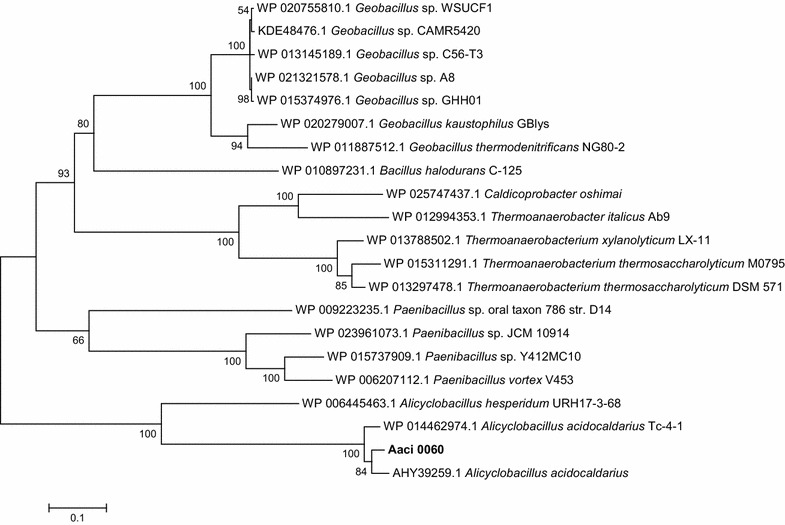
Phylogenetic tree showing distance of α-1,2-glucuronosidase encoded by gene locus Aaci_0060 in *A. acidocaldarius* strain DSM 446 genome to similarly annotated genes *Firmicutes*. Each gene in tree is listed by accession number and bacterium carrying gene

Phylogenetic analysis of the protein encoded by Aaci_0060 indicates relatedness to proteins from a number of other cellulolytic bacteria found in the phylum *Firmicutes* (Fig. 8). This protein from *A. acidocaldarius* shows greater than 50% homology to proteins that have been annotated as α-glucuronidases in both aerobic and anaerobic bacteria in the genera *Bacillus*, *Clostridium*, *Paenibacillus*, *Geobacillus*, and *Thermoanaerobacterium*.

Regulation of gene loci Aaci_0786, Aaci_0789, and Aaci_0797 was also stimulated during this experiment. The first of these genes, Aaci_0786, has been annotated as a glycoside hydrolase clan GH-D protein, specifically an α-galactosidase. A conserved domain comparison with other glycoside hydrolases in GenBank indicates specific hits to melibiase, with nonspecific hits to glycoside hydrolase family 31 (GH31) enzymes. Enzymes in the GH31 superfamily are present in all three domains of life, and activities include α-glucosidase, α-xylosidase, 6-α-glucosyltransferase, 3-α-isomaltosyltransferase, and α-1,4-glucan lyase. While this enzyme has been annotated as an α-galactosidase, and could be involved in removal of galactose from the xylan backbone of WAX, expression of this enzyme during growth on WAX may also indicate α-xylosidase activity.

**Fig. 9:**
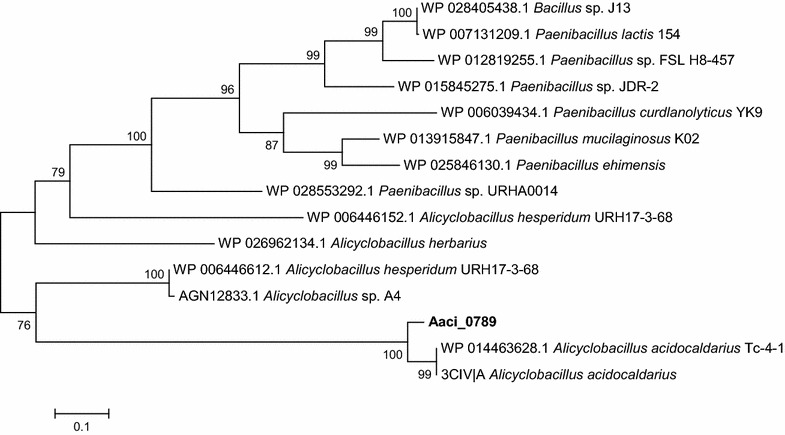
A phylogenetic distance tree of the glycoside hydrolase encoded by Aaci_0789, and homology to an endo-β-1,4-mannanase from another *A. acidocaldarius* strain, and the endo-β-1,4-xylanase from a number of other *Firmicutes*. Each gene in tree is listed by accession number and bacterium carrying gene

Protein sequence analysis and classification (IPR013781) of Aaci_0789 indicates the presence of a catalytic TIM beta/alpha barrel common to many different families of glycoside hydrolases. This protein is greater than 50% identical with hypothetical proteins containing glycoside hydrolase catalytic domains found in other species of *Alicyclobacillus*. The proposed protein product for Aaci_0789 shows homology to an endo-β-1,4-mannanase that was characterized in *Alicyclobacillus* species A4 and *A. acidocaldarius* strains Tc-4-1 and Tc-12-31 [26, 45] (Fig. 9). The glycoside hydrolase encoded by this gene locus also demonstrates up to 50% homology to endo-β-1,4-xylanases from a number of other *Firmicutes*, as well as hypothetical proteins from *Firmicutes* known to degrade xylan. In silico analysis of this gene along with expression results indicate a possible broad substrate range for this enzyme that would include mannose- and xylose-containing polysaccharides.

The final gene locus in this group, Aaci_0797, has been annotated as a glycoside hydrolase family 4, α-galactosidase. The protein encoded by Aaci_0797 is 99% identical to a β-galactosidase cloned from *A. acidocaldarius* [19]. This recombinant enzyme hydrolyzed *o*-nitrophenyl-β-d-galactopyranoside and *o*-nitrophenyl-β-d-fucopyranoside, but did not catalyze hydrolysis of *o*-nitrophenyl-β-d-glucopyranoside, *o*-nitrophenyl-β-d-xylopyranoside, or *o*-nitrophenyl-β-d-arabinopyranoside. These results indicate that Aaci_0797 is likely a β-galactosidase active toward galactose moieties on side chains of the WAX. Aaci_0797 also has greater than 70% homology to α-galactosidase from a number of other Gram-positive bacteria, including numerous species of *Paenibacillus*.

**Fig. 10:**
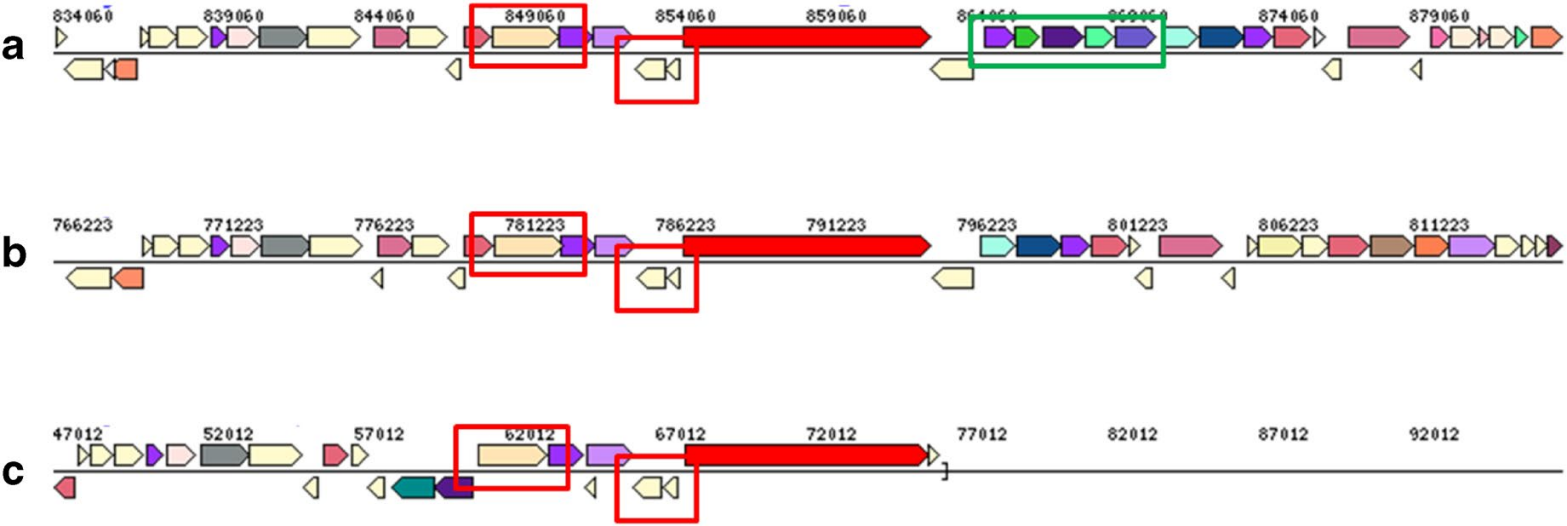
Genome neighborhood showing conserved genes encoding glycoside hydrolases (red boxes) in two *A. acidocaldarius* species, and *A. hesperidum* species URH17-3-68. Green box indicates genes for a glycoside hydrolase, an ABC-transporter and a transcriptional regulator, which are present in the *A. acidocaldarius* strain DSM 446 genome but not in *A. acidocaldarius* strain Tc-4-1. **a** *A. acidocaldarius* subsp. *acidocaldarius* (DSM 446), **b** *A. acidocaldarius* subsp. *acidocaldarius* (Tc-4-1), and **c** *A. hesperidum* URH17-3-68

Arrangement of the genes encoding the glycoside hydrolase clan GH-D (Aaci_0786) and the hypothetical protein containing a glycoside hydrolase catalytic domain (Aaci_0789) are nearly identical in *A. acidocaldarius* subsp. *acidocaldarius* (DSM 446), *A. acidocaldarius* subsp. *acidocaldarius* (Tc-4-1), and *A. hesperidum* URH17-3-68 (Fig. 10). However, this gene arrangement is not conserved in *A. herbarius* DSM 12609. DSM 446 appears to have a cassette containing the glycoside hydrolase family 4 α-galactosidase (Aaci_0797), genes encoding ABC-transporter components (Aaci_0794-0796), and a LacI family transcriptional regulator (Aaci_0793) that is not present in the Tc-4-1 genome. Two β-galactosidase genes can be found in the Tc-4-1 genome, but translated protein sequences show low homology to Aaci_0797, indicating that Tc-4-1 may lack α-galactosidase activity.

Annotation within the JGI-IMG/M categorizes Aaci_1218 with a product name of a glycoside hydrolase family 2 TIM barrel. Analysis of the protein sequence showed no signal sequence, indicating activity inside the cell. Gene Ontology (GO:0004565), clusters of orthologous groups (COG3250), and KEGG Orthology (KO:01190) indicate that this protein may be a β-galactosidase/β-glucuronidase. This 1041 amino acid protein shows the highest homology to glycoside hydrolases found in other *Bacilli.* This enzyme most likely acts on either galactose or 4-*O*-methyl-d-glucuronic acid side chains found in WAX. These results indicate that *A. acidocaldarius* was expressing genes related to side chain hydrolysis of the WAX, which were regulated upon addition of xylose. Aaci_1218 appears to be in an apparent operon with genes for an ABC-transport system and a LacI transcriptional regulator, all of which were highly downregulated.

**Fig. 11:**
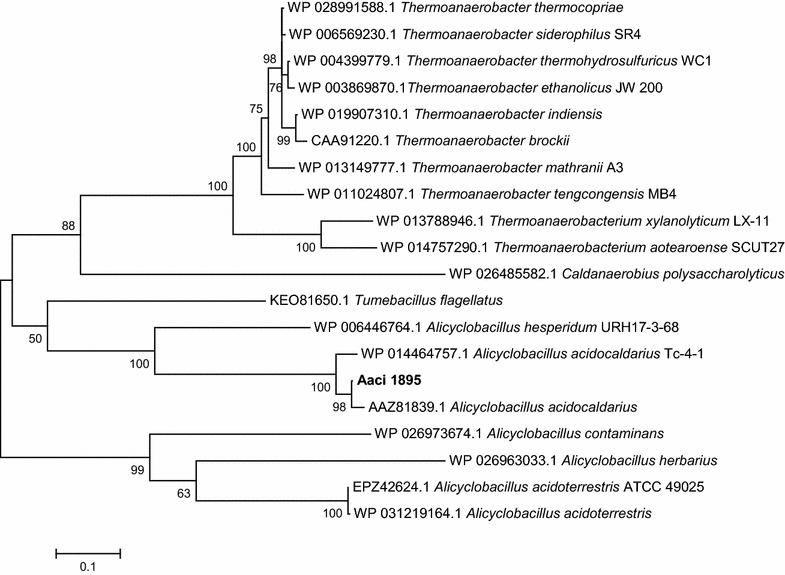
A phylogenetic distance tree showing glycoside hydrolase encoded by Aaci_1895, and homology to a β-galactosidase/β-glucosidase from other *Firmicutes*, as well as a number of *Thermoanaerobacter* species. Each gene in tree is listed by accession number and bacterium carrying gene

**Fig. 12:**
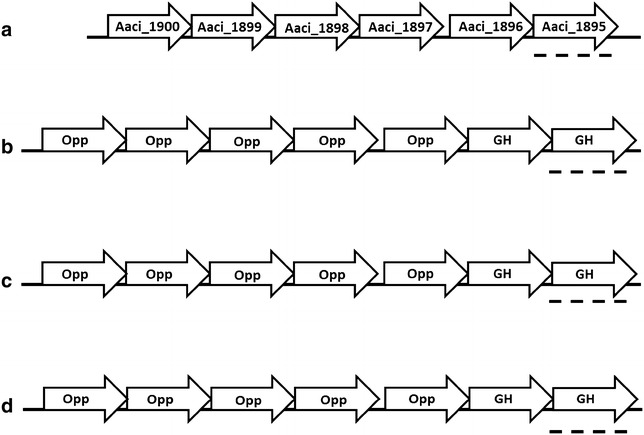
Schematic of the structure of an apparent operon associated with a β-galactosidase/β-glucosidase gene (Aaci_1895) in *A. acidocaldarius* strain DSM 446, and comparison to a similar operon in a number of *Thermoanaerobacter* species. **a** *A. acidocaldarius* strain DSM 446, **b** *T. siderophilis* strain SR4, **c** *T. italicus* strain Ab9, **d** *T. ethanolicus* strain JW 200. Dashed lines show glycoside hydrolase bearing similarity to Aaci_1895. Opp indicates various components of annotated ATP-binding cassette transporter for dipeptides, and GH indicates glycoside hydrolases

The 453 amino acid *A. acidocaldarius* protein encoded by Aaci_1895 has greater than 60% homology to β-galactosidase/β-glucosidase enzymes found in other *Firmicutes*, namely numerous species of *Thermoanaerobacter* (Fig. 11). Di Lauro et al. [6] characterized a recombinant β-glycosidase from *A. acidocaldarius* that showed wide substrate specificity and was able to hydrolyze β-d-gluco-, -galacto-, and fucosides. The protein encoded by Aaci_1895 is 98% identical to the characterized β-glycosidase, and thus is likely the same protein. Like many other glycoside hydrolase encoding genes in the *A. acidocaldarius* genome, Aaci_1895 is arranged in an operon with an ABC-type transport system; however, this specific ABC-type transporter has been annotated as an oligopeptide/dipeptide transporter. Utilization of oligopeptide/dipeptide ABC-type transporters for carbohydrate transport is not uncommon in other thermophilic lignocellulose-degrading bacteria, including *Thermotoga maritima* and *Caldicellulosiruptor saccharolyticus* [46, 47]. This gene arrangement, including the oligopeptide/dipeptide ABC-type transporter genes, is also common in a number of *Thermoanaerobacter* species. The primary difference between *A. acidocaldarius* and the *Thermoanaerobacter* species is the presence of an additional gene, which encodes a family 2 glycoside hydrolase, situated between the β-galactosidase/β-glucosidase and the transporter genes (Fig. 12). The family 2 glycoside hydrolase gene encoded in the *Thermoanaerobacter* genomes may have a similar function to the family 2 glycoside hydrolase encoded by Aaci_1218, which was also highly downregulated in *A. acidocaldarius*, as discussed above. Comparing the family 2 glycoside hydrolase from *Thermoanaerobacter mathranii* to the *A. acidocaldarius* subsp. *acidocaldarius* DSM 446 genome yielded the closest homology to Aaci_1218 (*E* value = 9e−10); other proteins had *E* values greater than 1.

**Fig. 13:**
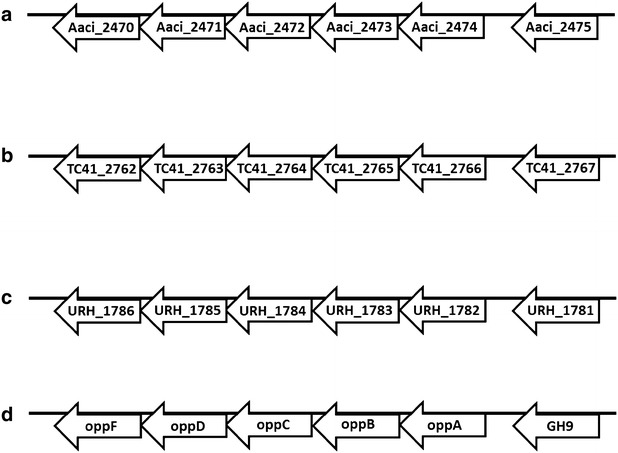
Schematic showing apparent operon structure associated with Aaci_2475, a gene encoding an endoglucanase, and comparison to other *Alicyclobacillus* species. **a** *A. acidocaldarius* strain DSM 446, **b** *A. acidocaldarius* strain Tc-4-1, **c** *A. hesperidum* strain URH17-3-68, **d** general operon structure showing proposed components associated with gene loci for each bacterium

Domain analysis of the 537 amino acid protein encoded by Aaci_2475 indicates that the enzyme may have endoglucanase activity, but since it appears to be active on WAX, the enzyme may have an expanded substrate range, and act on substrates other than cellulose or cellobiose. Endoglucanases with broad substrate specificity, including activity toward xylans, have been demonstrated in other thermophiles, including *Pyrococcus furiosus* and *T. maritima* [48, 49]. The glycoside hydrolase encoded by Aaci_2475 is next to an ABC-type oligopeptide transport system, which was also highly downregulated when xylose was added, as was the case with Aaci_1895 and neighboring genes. Analysis of the gene neighborhood where this family 9 glycoside hydrolase is found indicates that this apparent operon structure is common in numerous *Alicyclobacillus* species, including *A. acidocaldarius* subsp. *acidocaldarius* (DSM 446), *A. acidocaldarius* subsp. *acidocaldarius* (Tc-4-1), and *A. hesperidum* URH17-3-68 (Fig. 13). Xylan-hydrolyzing activity by an endoglucanase is not unprecedented; Hall et al. [50] characterized an endoglucanase from *Clostridium thermocellum* with xylanase activity. More recently, a bifunctional xylanase–glucanase from *Paenibacillus* sp. Strain E18 was characterized and showed activity toward xylan and glucan [51]. Likewise, bifunctional xylanase/endoglucanase enzymes have been generated from the metagenome libraries from a yak and bovine rumen microbial community and showed activity toward a variety of xylans and glucans [52, 53].

**Fig. 14:**
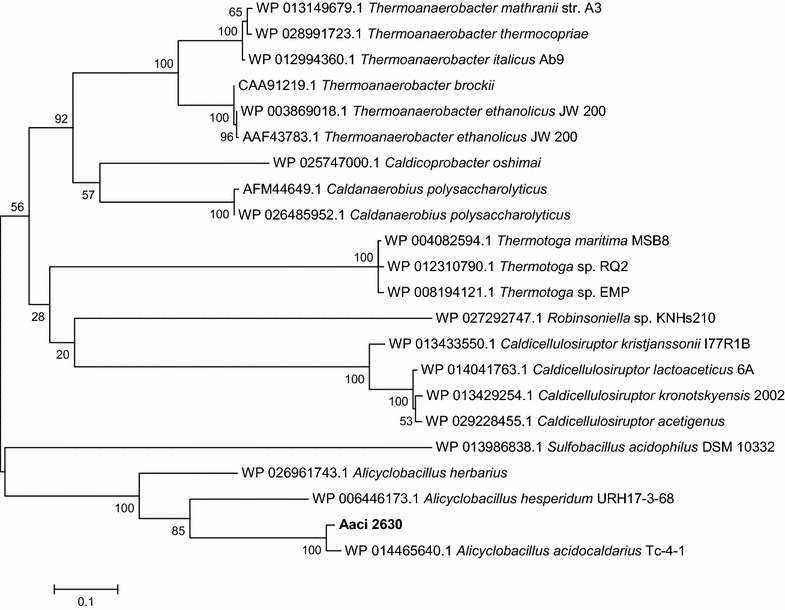
A phylogenetic distance tree showing glycoside hydrolase encoded by Aaci_2630, and homology to a β-xylosidase enzymes from other thermophilic lignocellulose degrading bacteria. Each gene in tree is listed by accession number and bacterium carrying gene

Aaci_2630 encodes a glycoside hydrolase family 3 domain protein with β-glucosidase or β-xylosidase activity. Regulation during growth on WAX further supports the possibility of β-xylosidase activity. Homology analysis of Aaci_2630 to other β-xylosidases genes deposited in the National Center for Biotechnology Information (NCBI) database shows greater than 50% similarity to family 3 glycoside hydrolases from other thermophilic, lignocellulose-degrading bacteria, including *Thermoanaerobacter*, *Caldicellulosiruptor* and *Thermotoga* species (Fig. 14). This proposed β-xylosidase also has ~ 60% identity with three enzymes expressed from recombinant DNA showing specificity toward xylo-oligosaccharides for which activity assays have been performed [54–56]. Xyl3A from *Caldanaerobius polysaccharolyticus* demonstrated both β-glucosidase and β-xylosidase activity, but activity toward xylooligosaccharides was the highest [55]. Enzymes that showed homology to Aaci_2630 from two species of *Thermoanaerobacter* have also been characterized as β-xylosidases. A bifunctional xylosidase–arabinosidase (XarB) from *T. ethanolicus* JW200 demonstrated activity toward a variety of substrates, but the highest activity/affinity was toward xylo- and arabinopyranoside molecules [56]. Similarly, a xylodextrin-hydrolyzing xylo-β-glucosidase from *T. brockii* was shown to possess both β-glucosidase and β-xylosidase activity [54]. Homology of Aaci_2630 with these other proteins that have demonstrated β-xylosidase activity and the downregulation in these experiments support classification of the encoded enzyme as a β-xylosidase.

**Fig. 15:**
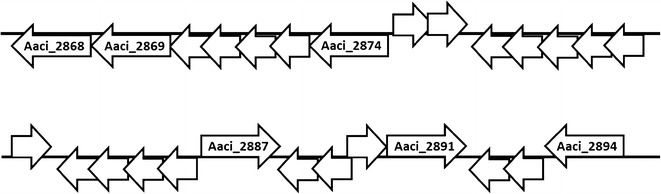
Schematic showing glycoside hydrolase genes from the *A. acidocaldarius* strain DSM 446 genome spanning DNA coordinates 2,926,031 to 2,962,624. Short arrows without gene locus are shown to demonstrate the numbers of genes between each glycoside hydrolase and directionality of genes

A section of the *A. acidocaldarius* genome from gene locus Aaci_2868 through Aaci_2894 appears to be a ‘hot spot’ for glycoside hydrolase activity because 6 of the 19 glycoside hydrolase encoding genes in *A. acidocaldarius* are found in this region (Fig. 15). Annotation in this region indicates genes encoding both cellulose- and hemicellulose-hydrolyzing enzymes. Half of the glycoside hydrolase genes were regulated during the experiment, maintaining the trend of higher regulation when xylose was added to the exponentially growing culture of *A. acidocaldarius*.

The translated protein from Aaci_2874 shows over 99% homology to an enzyme cloned from *A. acidocaldarius* that had α-amylase and pullulanase catalytic activity [20]. A thermoacidophilic α-amylase expressed from *Alicyclobacillus* sp. A4 which showed 65% identity with the protein encoded by Aaci_2874 also showed high activity toward starch compounds, but the substrate range of the enzyme was not determined [57]. Aaci_2874 is located in what may be an operon containing two other glycoside hydrolases—Aaci_2868 and Aaci_2869—neither of which was regulated to the extent of Aaci_2874 when xylose was added. Since there have been no reports of bifunctional α-amylases, the relationship of this enzyme to WAX depolymerization is not known, but regulation of this gene when *A. acidocaldarius* was grown on WAX indicates that this enzyme has adapted for hydrolysis of xylan polysaccharides.

During metabolism of WAX, α-arabinofuranosidase catalyzes the hydrolysis of terminal α-arabinofuranosidic linkages to xylose residues in the xylan backbone. Gene locus Aaci_2894 encodes a putative α-arabinofuranosidase that is associated with an operon involved in arabinose metabolism, for which all genes were downregulated, regardless of whether xylose or glucose was used as the inducing sugar. The protein product from this gene has greater than 60% homology to α-arabinofuranosidases found in a number of Gram-positive bacteria, including *Geobacillus*, *Paenibacillus,* and *Thermoanaerobacterium*. As with other genes previously discussed, this or similar operons appear to be conserved among *Alicyclobacillus* species.

**Fig. 16:**
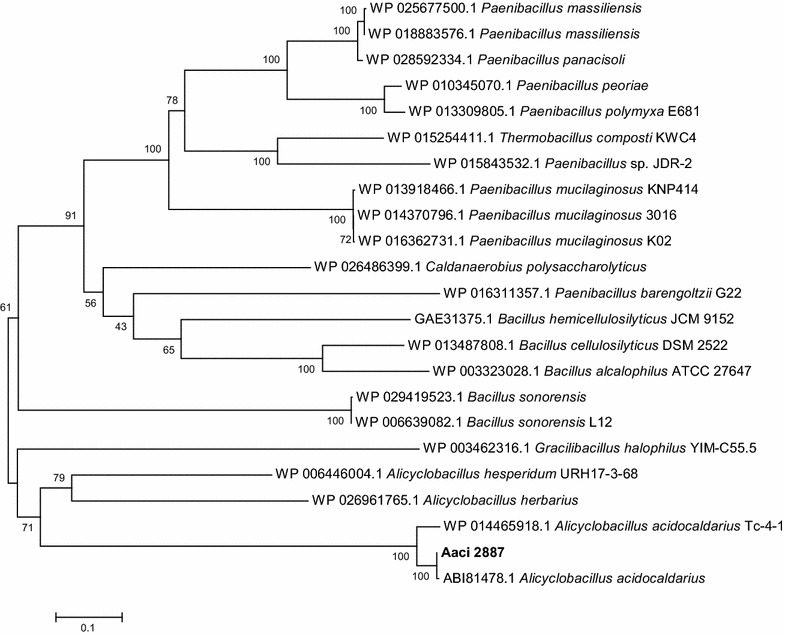
A phylogenetic distance tree showing glycoside hydrolase encoded by Aaci_2887, and homology to a α-xylosidase/α-glucosidase enzymes from other lignocellulose degrading *Firmicutes*. Each gene in tree is listed by accession number and bacterium carrying gene

Domain analysis for the protein encoded by Aaci_2887 is consistent with α-xylosidase/α-glucosidase activity, which consists of cleaving the terminal carbohydrate moiety from a variety of substrates. Since the gene was upregulated upon the addition of xylose, this 779-amino acid glycoside hydrolase may be involved in xylose metabolism, or even cleaving xylose that is linked to other sugars present in WAX. Comparison to other genes in the NCBI database indicates that Aaci_2887 is 50% identical to α-xylosidase/α-glucosidase enzymes in other Gram-positive lignocellulose utilizing bacteria, including numerous *Paenibacillus* and *Clostridium* species (Fig. 16). The protein sequence from Aaci_2887 is 99% identical to an enzyme expressed from recombinant DNA, thermostable α-glucosidase (ABI81478.1) from *A. acidocaldarius* that was deposited into GenBank, but no information on enzyme activity or specificity has been published. Other glycoside hydrolase family 31 enzymes that display α-xylosidase activity have been found in the Crenarchaeota *Sulfolobus solfataricus* (i.e., XylS) and the Gram-positive *Cellovibrio japonicus* [58, 59].

## Conclusions

In general, *A. acidocaldarius* appears to have enzymes necessary for the efficient depolymerization of WAX. Likewise, these enzymes were expressed when *A. acidocaldarius* strain ATCC27009 was grown on WAX. A number of the enzymes also appear to have a broad substrate range, allowing for hydrolysis of various bonds present in WAX even though the annotated activity of the enzyme would not predict this hydrolysis.

Glycoside hydrolases, for the most part, appear to be found in clusters, throughout the *A. acidocaldarius* genome. Not all of the glycoside hydrolase genes found at loci within these clusters were regulated during the experiment, indicating that a specific subset of the 19 glycoside hydrolase genes found in *A. acidocaldarius* were used during metabolism of WAX. While specific functions of the glycoside hydrolases was not tested as part of the research discussed, many of the glycoside hydrolases found in the *A. acidocaldarius* Type Strain appear to have a broader substrate range than represented by the glycoside hydrolase family in which the enzymes were categorized.

## Materials and methods

### Inoculum development

*Alicyclobacillus acidocaldarius* ATCC 27009 was purchased from the American Type Culture Collection (ATCC) and used for all experiments. Chemostat inoculum was prepared by inoculating 1 mL of the frozen stock into 25 mL of Modified 402 Medium, which contained the following (g/L): (NH_4_)_2_SO_4_ (1.3), Fe(III) EDTA (0.047), CaCl_2_·2H_2_O (0.07), MgSO_4_·7H_2_O (0.25), KH_2_PO_4_ (3.0), and xylose (4.0). In addition, 1 mL of a mineral (solutions A and B) and vitamin stock (solution C) were added. Solution A contained (g/L): MgCl_2_ (25), CaCl_2_·2H_2_O (6.6), H_3_BO_3_ (0.58), FeCl_3_·6H_2_O (5), Co(NO_3_)_2_·6H_2_O (0.05), NiCl_2_·6H_2_O (0.02). Solution B contained (g/L): MnSO_4_·H_2_O (2.0), ZnSO_4_·7H_2_O (0.5), CuSO_4_·5H_2_O (0.15), Na_2_MoO_4_·2H_2_O (0.025). Solution C contained (g/L): pyridoxine hydrochloride (0.08), folic acid (0.012), thiamine hydrochloride (0.13), riboflavin (0.042), nicotinamide (0.084), *p*-aminobenzoate (0.088), biotin (0.01), cyanocobalamin (0.0004), d-pantothenic acid, calcium salt (0.086), *myo*-inositol (0.021), choline bromide (0.053), orotic acid, sodium salt (0.021), and spermidine (0.1). Base medium was autoclaved (121 °C, 20 psi) for 30 min prior to use; KH_2_PO_4_ solution was adjusted to pH 4.0, autoclaved separately, and added once the base medium had cooled. The 25 mL *A. acidocaldarius* culture was grown overnight and then used to inoculate 250 mL of Modified 402 Medium containing 4 g/L xylose. This overnight culture grown at pH 4 at a temperature of 60 °C was then used to inoculate the chemostat.

### Chemostat studies

Experiments were performed in a BioFlo 3000 chemostat system (New Brunswick Scientific, Enfield, CT). Medium was added to the reactor, and the entire reactor was autoclaved at 121 °C, and a pressure of 20 psi for 1 h. WAX (Megazyme, Inc.) was prepared by first wetting 2 g with 95% ethanol. Once in a slurry, 190 mL of sterile distilled water (pH 4.0) was added, and the solution was heated for 30 min to evaporate residual ethanol. Pre-dissolved WAX (2 g/190 mL medium) was then added to the 2 L of Modified 402 Medium in the chemostat, giving a final WAX concentration of 1 g/L. The chemostat was run in batch mode and the temperature was held at 60 °C and the pH of the growth medium was automatically controlled to 4.0 using the addition of 1 N NaOH. To ensure that the cultures were not oxygen limited, dissolved oxygen was controlled to 10%.

Once the *A. acidocaldarius* culture reached an OD_600_ of 0.5 while growing on WAX, a sample was taken for RNA extraction, glucose or xylose was added at a concentration of 2 g/L, and then a second sample was immediately (within 5 min) taken for RNA extraction. Three biological replicates for each condition were performed.

### Isolation of total RNA

Samples were taken from the chemostat, and RNA was stabilized using RNA Protect Bacteria Reagent (Qiagen, Valencia, CA), followed by flash freezing of the cell pellet in liquid nitrogen and storage at − 80 °C. Total RNA was extracted from the *A. acidocaldarius* cells using an RNeasy Midi Kit (Qiagen, Valencia, CA) with slight modification of the manufacturer’s protocol. *A. acidocaldarius* cells were thawed, and lysis was accomplished by adding 200 µL of Tris–EDTA buffer containing 15 mg/mL lysozyme and 0.1 mg/mL proteinase K. Samples were vortexed for 10 s and then incubated at room temperature for 15 min with shaking. Manufacturer’s instructions supplied with the kit were followed up to elution of the RNA. To increase RNA yield, the flow through was re-applied to the spin filter and centrifuged. Residual DNA in the samples was removed by treatment with Ambion TURBO DNA-free kit (Life Technologies, Grand Island, NY). RNA was purified to remove compounds that might interfere with cDNA synthesis and concentrated using ethanol precipitation. To inhibit RNA degradation during storage, 1 µL of Ambion Superase-In RNase Inhibitor (Life Technologies, Grand Island, NY) was added. RNA concentration and purity were determined using a NanoDrop ND-1000 spectrophotometer (Thermo Scientific, Wilmington, DE). RNA integrity was determined using an RNA Nano Chip Kit run on an Agilent 2100 Bioanalyzer (Agilent, Santa Clara, CA).

### Synthesis of cDNA

Double-stranded cDNA was synthesized from total RNA using the Invitrogen Superscript Double-Stranded cDNA Synthesis Kit (Life Technologies, Grand Island, NY) according to manufacturer’s instructions, with the following modifications. Following cDNA synthesis, residual RNA was degraded by adding RNase A, followed by proteins removal using phenol:chloroform:isoamyl alcohol, and separation using Phase Lock Tubes (5 Prime, Inc., Gaithersburg, MD). cDNA in the aqueous phase was then precipitated and concentrated by ethanol precipitation. The pellet was dried and then resuspended in 20 µL of DNase/RNase-Free water and allowed to solubilize overnight. cDNA concentration in each reaction was determined using a NanoDrop ND-1000 spectrophotometer (Thermo Scientific, Wilmington, DE). A DNA 7500 Chip Kit run on an Agilent 2100 Bioanalyzer (Agilent, Santa Clara, CA) was used to verify that most of the cDNA was ≥ 400 bp.

### Microarray experiments and data analysis

Microarrays were designed and synthesized by NimbleGen using their 4 × 72 K Custom Gene Expression Array format from the complete genome sequence information for *A. acidocaldarius* ATCC 27009 (DSM 446). Seven probes, each 60 nt long, were designed for each of the 3554 identified open reading frames in the genome, based on a genome sequence generated by Integrated Genomics, Inc for the Idaho National Laboratory. Each probe was synthesized on the microarray in triplicate. Control probes were also included to ensure that there was no intra-quadrant contamination during the hybridization process.

One color cDNA labeling using Cy3, hybridization to the *A. acidocaldarius* microarrays, array imaging, and initial analysis of the array data were performed by NimbleGen. Data were normalized using Nimblescan software, which normalizes probe response using quantile normalization and gene calls generation using Robust Multichip Averaging (RMA) [60, 61]. Log_2_-transformed RMA data files were imported into ArrayStar 4 software (DNASTAR, Inc., Madison, WI), and the mean expression levels of three replicate arrays for each condition were considered. For comparison of gene expression, statistical significance was determined with a Bonferroni corrected moderate *t* test, and only genes where expression had greater than 95% confidence (*p* ≤ 0.05) were considered significant.

Comparative analysis of the *A. acidocaldarius* genome was performed using the Integrated Microbial Genomes feature within the Joint Genome Institute [62]. Homology determinations for *A. acidocaldarius* proteins were accomplished using the Basic Local Alignment Search Tool (BLAST) for protein sequences using the ‘blastp’ algorithm [63, 64]. The nonredundant protein sequence database was used for searches, and uncultured and environmental sample sequences were excluded from the search. Phylogenetic trees comparing protein sequences were generated using the Molecular Evolutionary Genetics Analysis (MEGA6) software program [65]. Protein sequences were aligned using the MUSCLE program within MEGA6 [66, 67]. Phylogenetic reconstruction was accomplished using the maximum likelihood statistical method, and distances between sequences were determined using 1000 bootstrap replicates.

## Abbreviations

WAX: wheat arabinoxylan
NCBI: National Center for Biotechnology Information
CCR: carbon catabolite repression
PTS: phosphotransferase system
ATP: adenosine triphosphate
ABC: ATP binding cassette
ATCC: American Type Culture Collection
CE: carbohydrate esterase
DSM: Deutsche Sammlung von Mikroorganismen
DNA: deoxyribonucleic acid
KEGG: Kyoto Encyclopedia of Genes and Genomes
JGI: Joint Genome Institute
IMG: Integrated Microbial Genomes
GO: gene ontology
COG: cluster of orthologous genes
BLAST: Basic Local Alignment Search Tool
GH: glycoside hydrolase
RMA: Robust Multichip Averaging
MEGA: Molecular Evolutionary Genetics Alignment
MUSCLE: Multiple sequence comparison by log-expectation
cDNA: complementary DNA
RNA: ribonucleic acid
